# Desiccation Cracking Behavior of Polyurethane and Polyacrylamide Admixed Clayey Soils

**DOI:** 10.3390/polym12102398

**Published:** 2020-10-18

**Authors:** Changqing Qi, Yuxia Bai, Jin Liu, Fan Bu, Debi Prasanna Kanungo, Zezhuo Song, Xilong He

**Affiliations:** 1 School of Earth Sciences and Engineering, Hohai University, Nanjing 210098, China; qichangqing@hhu.edu.cn (C.Q.); byxhhu@163.com (Y.B.); bf_hhu@163.com (F.B.); szzhhu@163.com (Z.S.); xilonghe96@163.com (X.H.); 2 Council of Scientific and Industrial Research (CSIR)-Central Building Research Institute (CBRI), Roorkee 247667, India; debi.kanungo@gmail.com

**Keywords:** clayey soil, polymer, evaporation, desiccation cracking, microstructure

## Abstract

There has been a growing interest in polymer applied for soil reinforcement in recent years. However, there little attention has been paid to the effects of polymer on soil cracking behavior, and cracks significantly change soil strength and hydraulic properties and alter reinforcement effectiveness. This study investigated the desiccation cracking behavior of polyurethane (PU) and polyacrylamide (PAM) admixed clayey soils with different polymer concentrations by performing desiccation cracking tests. Scanning electron microscope (SEM) observation was also carried out to obtain the internal structure of these soils. The results show that PU and PAM addition both prolonged the initial evaporation stage, accelerated later evaporation processes, and the effects were related to polymer concentration. Final cracks morphology analyses show that PAM addition slightly reduced the cracking and crushing degree and kept the soil relatively intact, while PU addition slightly enhanced the cracking and crushing degree of soil. In addition, PU and PAM addition both increased the width and length of cracks. The scanning electron microscopy (SEM) analyses show that the effects of polymer on soil evaporation and cracking could be concluded as: (1) storing water in voids, (2) influencing water immigration channel, (3) providing space for soil shrinkage, and (4) enhancing the connection between aggregates, which did not fully come into play because of the existence of hydrogel form. These achievements provide a certain basis for the research of desiccation cracking behavior of polymer treated soil and make significant sense for the safe and effective running of related projects.

## 1. Introduction

Polymer has gained increasing attention for soil reinforcement (i.e., improving soil poor performances such as cohesion and ductility) in recent years [1,2,3] because of its excellent characteristics in terms of efficient performance, better sustainability, providing a clean and healthy environment, and ease of operation [4,5,6]. The mechanism of polymer reinforcement soil is reported whereby the polymer is strongly absorbed on the surface of soil grains via physio-chemical force, and subsequently enwraps grains and interlinks them together by their molecules uncoiling [7,8], which in turn enhances soil stability. Many researchers have studied the behaviors of polymer treated soil in the laboratory and concluded that polymer treatment can effectively enhance soil mechanical properties in terms of compressive strength, shear strength, and tensile strength as well as ductility [9,10,11,12], also improving soil hydraulic properties in terms of water hold ability, anti-erodibility, and permeability [13,14]. Moreover, some polymers have been successfully used in different geotechnical engineering applications, such as: stabilization treatment of clay slope topsoil [15], enhancing the strength of weak soil [6], injection for pavement slab stabilization [16], and pavement subgrade remediation [17]. However, further examination shows that previous studies mostly investigate the effects of polymer on the mechanical and hydraulic performances of soil. Furthermore, there are few studies on the behavior of desiccation cracking of polymer treated soil [18]. 

Cracking is a common natural phenomenon during soil drying that results from water loss by evaporation. In general, the formation and increase of cracks alter soil performance, including mechanical and hydraulic properties, which consequently leads to damages for buildings and geotechnical engineering [19,20]. Indeed, a soil mass was split into detached clods by the cracks, and this in turn weakened the integrity and strength of the soil [21,22]. On the other hand, hydraulic properties of soil are significantly influenced by these cracks, considering that the flow rate and velocity are controlled by the size and tortuosity of cracks, while the distribution and the connectivity of these cracks determine the flow pathway [23]. Rayhani et al [24] indicated that cracks could lead to an increase on the hydraulic conductivity of clay soil by 12–34 times. In field projects, the cracks observed on the surface of a clayey soil slope could provide a channel for rainwater entering into a deeper level of the slope, and consequently decrease the strength of the soil, which may cause damage to the slope [25,26]. Also, the cracks observed in the landfill clay liners could significantly accelerate water infiltration into clay liners, and consequently weaken the efficiency of clay liners [27,28]. Owing to these vital effects of desiccation cracks and the wide application of polymer in different geotechnical projects, focusing on the cracking behavior of polymer treated soils makes significant sense for the safe and effective running of related projects.

Considering the effects of soil desiccation cracks, the parameter acquisition and quantitative analysis of cracks are of great significance to the study of soil cracking [29,30]. Zein el Adedine and Robinson [31] used the number of intercepts between a transect and crack to estimate the length of cracks. Miller et al. [32] introduced the crack intensity factor (CIF) as a descriptor of the extent of surface cracking, which was defined as the ratio of cracks area to the total surface area of a drying soil mass. However, the original crack pattern is often disturbed by human activities and equipment, which results in large measurement errors. With the continuous improvement of computer computing ability and the continuous development of image processing technology and software, it has gradually become a research hotspot to use computer to process and calculate fracture images and obtain relevant crack parameters [32,33,34]. However, because the soil is a mixture with complex structure, its cracking is affected by many factors. After mixing with polymer, the structure of soil is changed, and the cracking of soil is also affected. The existing research is insufficient, so it is of great significance and value to study the cracking of polymer mixed soil by using fine image processing software.

Given these considerations, this study investigated the desiccation cracking behavior of two different commonly used polymers (polyurethane and polyacrylamide) admixed with clayey soils. The effects of polymer concentration were also considered. A series of desiccation tests were conducted, and the water evaporation, crack initiation, and propagation processes were recorded during drying. With the aid of an image-processing technique, the geometrical and morphological characteristics of a crack pattern were analyzed quantitatively. In addition, the microstructure changes were also studied using scanning electron microscopy.

## 2. Materials and Methods

### 2.1. Materials

A clayey soil derived from Nanjing area of eastern China, widely distributed in middle and lower reaches of Yangtze River is used in present study. Its physical parameters were measured according to the national criterion for geotechnical tests in China (i.e., GB/T 50123–1999), and the results are presented in Table 1. 

**Figure 1:**
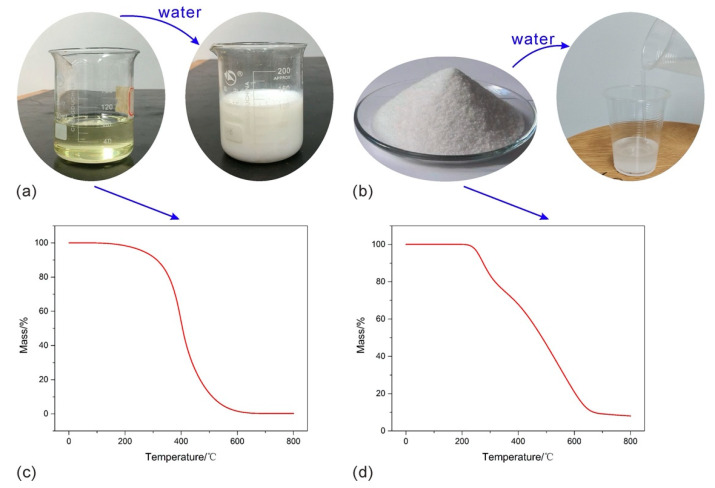
The polymer used in this study: (**a**) PU; (**b**) PAM; (**c**) thermogravimetric result of PU; (**d**) thermogravimetric result of PAM.

Polyurethane and polyacrylamide are used in this study because of their wide application as soil conditioners, enhancing aggregates stability, declining soil erosion, and improving soil mechanical properties [35,36,37]. The polyurethane used herein is prepared by the polymerization of poly-oxypropylene diol (PPG, Jining Hongming Chemical Reagent Co., Ltd., Jining, China), poly-oxyethylene glycol (PEG, Shanghai Ika Biotechnology Co., Ltd., Shanghai, China), and toluene diisocyanate (TDI, Nantong Runfeng Petrochemical Co., Ltd., Nantong, China), and exhibited as a light-yellow transparent emulsion (Figure 1a). The PU could dissolve in water, emulsify, and disperse, and finally form a stable system (Figure 1a). It has a viscosity of 650−800 MPa∙s, specific gravity of 1.15, solid content higher than 88%, and pH of 7, and contains a variety of functional groups such as –OH, −NCO. Additionally, thermogravimetric analysis was carried out with a heating rate of 10 °C/min, a heating range of 0–800°C, and the results are shown in Figure 1c.

An anionic polyacrylamide provided by Henan Liansheng Environmental Protection Technology Co., Ltd., Gongyi, China is used. This PAM is polymerized by high purity acrylamide monomer, pure water, initiator, and corresponding auxiliaries in a suitable pH, temperature, and nitrogen environment. After that, the colloidal substance is granulated, dried, and crushed, and the solid PAM was obtained. This white power has a high-molecular-weight of 8–12 × 10^6^ Da and 20% of hydrolysis, also its solid content is higher than 88%. Note that the PAM solution was obtained by dissolving in tap water rather than deionized water, to enhance the dissolution of PAM [38]. The used PAM and its dissolved solution are shown in Figure 1b. Thermogravimetric analysis for PAM was also carried out with a heating rate of 10 °C/min, a heating range of 0–800 °C, and the results are given in Figure 1d.

### 2.2. Sample Preparation and Test method

The craw clayey soil was air-dried, crushed, and sieved at 2 mm in the laboratory. For the reference sample, the initial saturated slurry was prepared by thoroughly mixing dry soil powder and water. The water content of slurry is 60%, which is much higher than its liquid limit. For admixed samples, the polymer was dissolved in water first, followed by adding into dry soil and thoroughly stirring. The desired mixture was then transferred into a plastic container with an inner dimension of 10 × 10 × 5 cm (length × width × height). Afterwards, the mixture was further homogenized with the aid of a mechanical device lasting for 3–5 min. The container was carefully vibrated to remove the entrapped air bubbles in the mixture and the surface of slurry was flattened. The final mixture was then conserved at room condition for 2 days for curing. The final thickness of the slurry was about 12 mm. The prepared sample was left to evaporate at constant room temperature about 20 ± 2 °C and the relative humidity of 35% ± 3%. During evaporation process, the variation of sample weight was recorded by an electronic balance with an accuracy of 0.01 g, and the initiation and propagation of cracks was photographed by a digital camera for further analyses.

The polymer concentration was set as 0.25%, 0.5%, 0.75%, and 1% for PU, and 0.025%, 0.05%, 0.075%, and 0.1% for PAM, according to their consistency and viscosity characteristics as well as reinforcement efficiency. 

### 2.3. Image Processing and Quantitative Analysis

**Figure 2:**
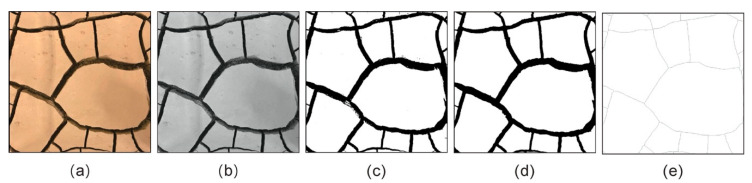
The procedure of cracks image processing: (**a**) original image; (**b**) grey image; (**c**) binary image; (**d**) image de-noising; (**e**) crack skeletonization.

The technique of digital image processing is usually applied to obtain quantitative and accurate data for further analyses of cracking behavior [23,39]. In this study, the digital image-processing technique developed by Nanjing University and introduced by Tang et al. [23] and Liu et al. [40] was used. The procedure is presented in Figure 2 and could be described as: transferring original color image to grayscale → image binarization → image de-noising → crack skeletonizing. Based on the difference of gray level in the images and cluster analysis method, the experimental soil cracking images are processed to form binary images of cracks, as shown in Figure 2c. The black area represents the cracks, and the white area represents the soil clods. As shown in Figure 2d, closed operations are used to repair individual fracture gaps and remove the fine spots. For the crack area, the nodes of the crack can be determined according to the number of surrounding pixels. By tracing and identifying the adjacent nodes, the median axis of the crack can be obtained, which is also regarded as the skeleton of the crack. The determination of crack intersection, length, and number are based on the skeleton network formed by the skeleton of the crack. Note that, only the central part of 9 × 9 cm is used (in Figure 2a) in order to eliminate container boundary effects [41].

The following quantitative parameters obtained from these processed images were determined and calculated [23,25,41]:

- Crack intensity factor (CIF) refers to the ratio of plane cracks area to the total area of the sample. The higher the value of CIF, the greater the degree of surficial cracking.
- Number of nodes (*N*_n_) and number of cracks (*N*_c_). The skeleton line between two adjacent nodes on the skeleton is considered as a crack (Figure 2e). In general, *N*_n_ and *N*_c_ can reflect the cracked degree of sample surface.
- Average length of cracks (*L*_av_) and average width of cracks (*W*_av_). The crack length was determined by calculating the distance between intersections on the skeleton (Figure 2e). The crack width was determined by calculating the shortest distance from a randomly selected point on one boundary to the opposite boundary of the crack segment.
- Number of clods (*N*_cl_) and average area of clods (*A*_av_). The clod refers to the independent closed area which is split by crack, that is, the closed white area in Figure 2c,d.
- Probability density function (PDF) of crack length *f*(*L*), crack width *f*(*W*), and clods area *f*(*A*), which could help analyzing the distribution characteristics of crack length, width, and clod area from the statistical level and providing more reliable results. Setting the PDF of crack length *f*(*L*) as an example, it is defined as:
  (1) $$f\left(L\right)=\frac{\Delta N_{ci}}{N_{c}\cdot \Delta L}$$
  where *N_c_* is the total number of cracks and ∆*N_ci_* is the number of cracks whose length ranges between *L_i_* + ∆*L*. *f*(*L*) refers to the percentage of cracks number whose length ranges between *L_i_* + ∆*L* to the total cracks number. In this study, the ∆*L* = 5 mm, ∆*W* = 0.5 mm, ∆*A* = 100 mm^2^ was chosen for the PDF of *f*(*L*), *f*(*W*), and *f*(*A*) respectively.

### 2.4. Microstructure Observation 

The microstructure of the reference and admixed samples is investigated using SEM images for further understanding the soil evaporation process and cracking behavior. After the completion of evaporation, the selected sample was prepared as a cube with size of 0.5 cm × 0.5 cm × 2 cm, which was selected from the volume center of the sample. Subsequently, the cube was subject to dehydration and spraying gold treatments in turn. Then, the SEM observation was performed.

## 3. Results

### 3.1. Evaporation Characteristic

**Figure 3:**
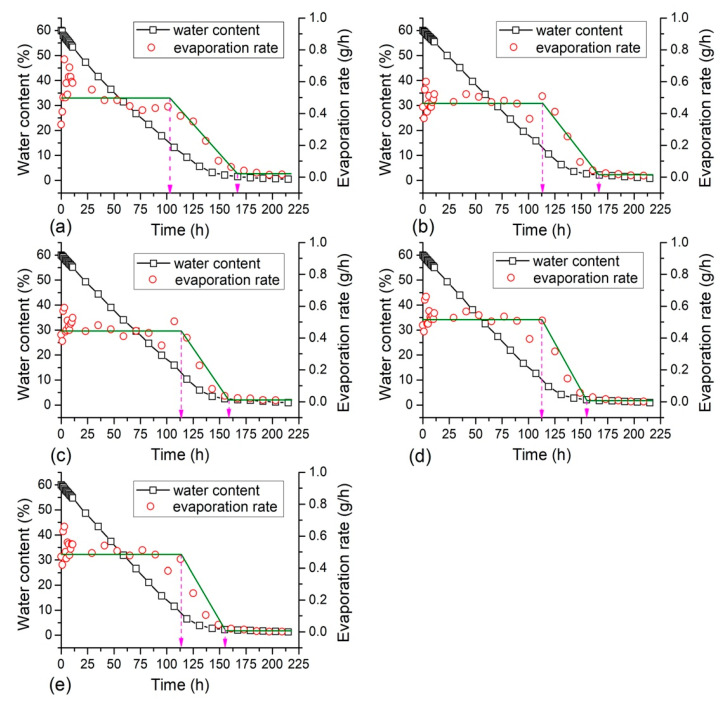
Evaporation process curve of PU admixed samples: (**a**) PU = 0%; (**b**) PU = 0.25%; (**c**) PU = 0.5%; (**d**) PU = 0.75%; (**e**) PU = 1%.

**Figure 4:**
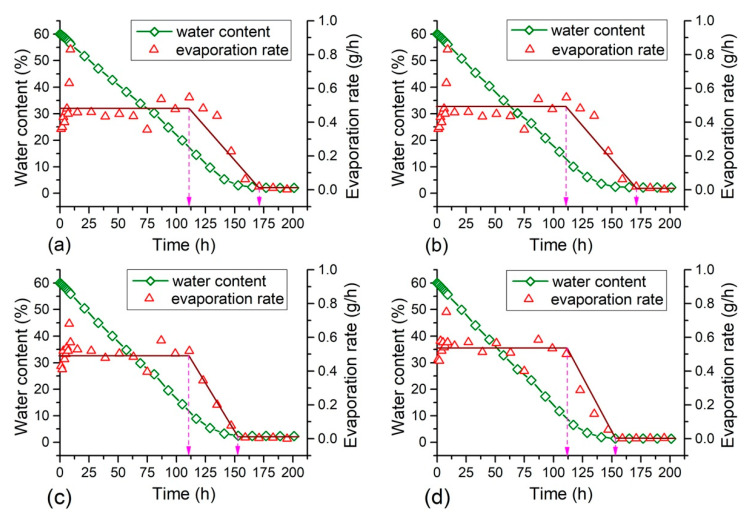
Evaporation process curve of PAM admixed samples: (**a**) PAM = 0.025%; (**b**) PAM = 0.05%; (**c**) PAM = 0.075%; (**d**) PAM = 0.1%.

The changes of water content and evaporation rate with time obtained from different samples are presented in Figure 3 and Figure 4. It is observed that the water content of each sample reduced proportionally with time at the beginning. After about 150 h, the evaporation slowed down and remained stable. Three different stages of evaporation process could be identified according to the evaporation rate: (1) the constant rate stage, (2) the deceleration rate stage and (3) the residual stage, which was consistent with the results reported by Zhang et al. [41] and Tang et al. [42]. It is found that the water loss mainly took place during the constant rate and the deceleration rate evaporation stages, while the evaporation rate tended to zero and the water content remained stable while it came into the third evaporation stage. 

It also appears from Figure 3 and Figure 4 that the soil evaporation process was affected by PU and PAM addition. For the sample with a small concentration of PU (Figure 3b), it took slightly longer time to come into the third residual stage in comparison with the reference sample (Figure 3a). However, for the sample with higher PU concentration of 0.5%, 0.75%, and 1%, the time became short which was obviously lower than 162.5 h (Figure 3c,d). In addition, the duration of the first evaporation stage of PU admixed sample (about 112.5 h) was significantly longer than that of the reference sample (about 100 h), because of its relatively low evaporation rate. In addition, the soil transformed from a saturated state to unsaturated once it came in to the deceleration rate evaporation stage [19]. These observations suggest that the PU addition could alter soil evaporation process to a certain extent. PAM addition showed similar effects on soil evaporation process as PU. The PAM addition with concentration of 0.025% and 0.05% prolonged the period of the first and second evaporation stages to about 175 h, while 0.075% and 0.1% PAM addition shortened this duration. Also, the PAM admixed samples with different concentrations took similar time for the constant rate evaporation stage (about 112.5 h), which was longer than that of the reference sample. It could be concluded that polymer addition with small concentration could delay soil whole evaporation process, and the addition with higher concentration prolonged the first evaporation stage but shortened the time came into the third residual stage. Moreover, there was no significant difference between PU and PAM effects on soil evaporation process. 

**Figure 5:**
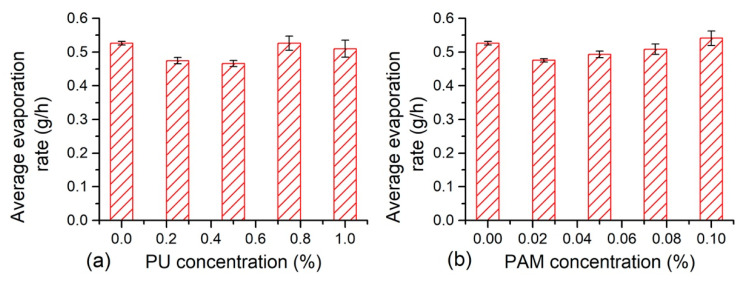
The average evaporation rate of (**a**) PU admixed samples and (**b**) PAM admixed samples.

It shows that the duration of the first evaporation stage could last for 100 to 112.5 h, the average evaporation rate during this stage is calculated for further understanding the effects of polymer on soil evaporation. The related results are displayed in Figure 5. The average evaporation rate of admixed samples increased gradually with polymer concentration, but was slightly smaller than that of clayey soil. The average evaporation rate of the reference sample was 0.53 g/h, and the PU and PAM addition both led to a reduction of the average evaporation rate to 0.47 g/h and 0.48 g/h respectively, i.e., decreased by 12%. The PU and PAM addition both delayed the initial evaporation process to a certain extent, and the effectiveness was dependent on the additive concentration. 

### 3.2. Crack Propagation and Pattern

**Figure 6:**
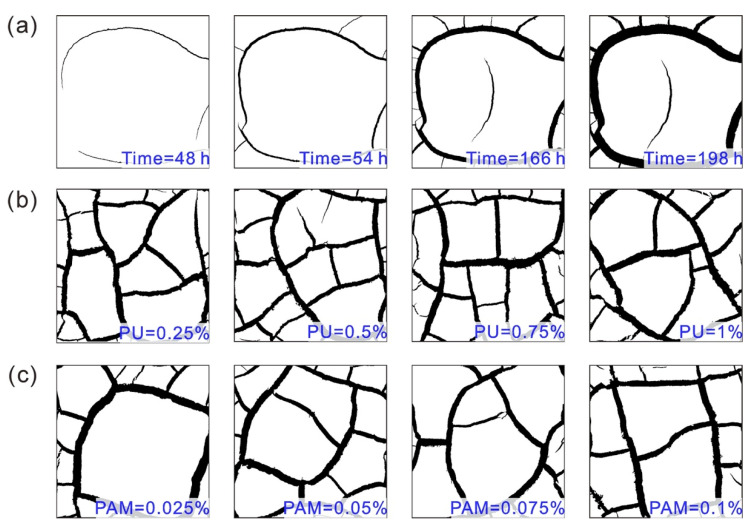
(**a**) Crack propagation during drying for clayey soil without additive over time; (**b**) The crack pattern of PU admixed samples; (**c**) The crack pattern of PAM admixed samples.

The crack propagation for reference sample over time is presented in Figure 6a. Note that the binary images are provided for more clearly revealing cracks propagation and pattern. It is observed that the cracking process of samples could be divided into three periods [23,42]: (1) initiation and growing of main cracks, (2) initiation and growing of sub cracks, and (3) relative stable period. During the initial period, it was found that the cracking usually started at some independent and random location of samples, flowed by growing randomly until ran into an existing crack. Such single cracks are considered as main crack, which was perpendicular to each other and the intersection angle tended to 90° and a “T” shape could be identified at the crack intersection. Most of cracks took place during the constant rate of evaporation stage when the soil was in saturated condition [19,41]. With further evaporation, some branch cracks (i.e., sub cracks) occurred at the random position on the main crack and grew perpendicular to the direction of the main crack, which was usually accompanied by main crack propagation. Tang et al. [43] explained that why the sub crack was generally perpendicular to the main cracks was because that crack propagation was a kind of tensile failure, and always grew towards the perpendicular to the direction of the maximum tensile stress. These sub cracks ended in the same way as the main cracks when they rejoin another existing main crack perpendicularly. Coming into the third stage, the crack network become stable and there was no formation of new cracks, which might have resulted from that the size of existing soil clods being smaller than its critical crackable size [23]. Moreover, the crack width increased at this period. Also, the final width of main cracks was greater than that of sub cracks.

The final crack patterns of PU and PAM admixed samples are displayed in Figure 6b,c, respectively. Comparing with the reference sample, they have the same cracking process, which also could be divided into three periods: initiation and growing of main cracks, initiation and growing of sub cracks, and a relative stable period. In addition, a “T” and “+” shape crack intersection was identified, and all the clods were exhibited as regular polygons. It also appears that both the PU and PAM additions had no obvious influences on crack pattern from a visual level, nor did the additives concentration. In contrast, the PU admixed sample possessed higher connectivity in comparison with the PAM admixed sample. Further analyses should be performed with the aid of the technique of image processing.

**Figure 7:**
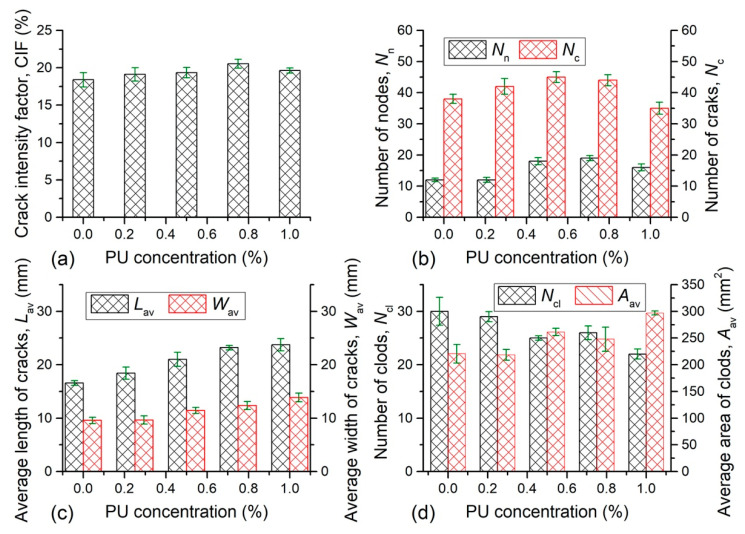
The quantitative analyses results on cracks of PU admixed sample: (**a**) crack intensity factor; (**b**) numbers of nodes and cracks; (**c**) average length and average width of cracks; (**d**) number of clods and average area of clods.

**Figure 8:**
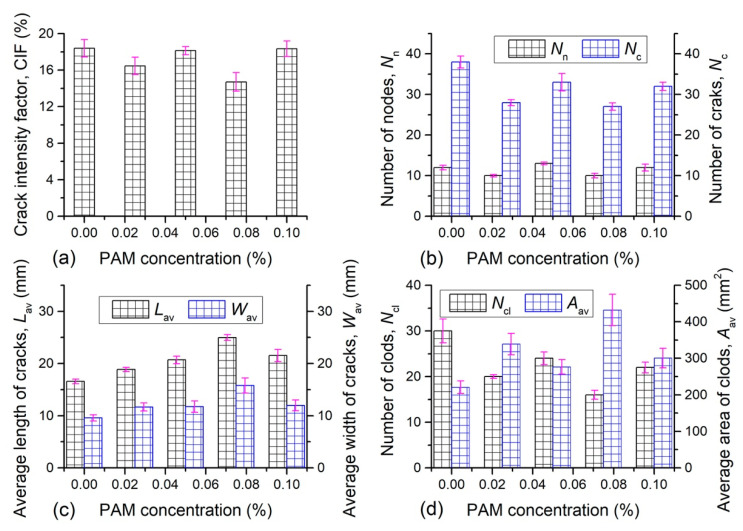
The quantitative analyses results on cracks of PAM admixed sample: (**a**) crack intensity factor; (**b**) numbers of nodes and cracks; (**c**) average length and average width of cracks; (**d**) number of clods and average area of clods.

The obtained crack patterns at the end of evaporation process from each sample were quantitatively analyzed by image-processing technique, and the related results were presented in Figure 7 and Figure 8. Figure 7a and Figure 8a display the CIF of different samples. The CIF of natural soil was 18.39%, suggesting that 18.39% of sample surface was covered by cracks. For PU admixed samples, the CIF increased from 19.12% to 20.53% with different concentration, which was slightly higher than that of the reference sample. However, the CIF fell between 14.71% and 18.33% with PAM addition, indicating that the sample surface was covered with smaller cracks in comparison with natural soil. In the current study, it was observed that the reference sample surface generated 38 cracks and 12 intersections, and was split into 30 soil clods during drying. At the same condition, the number of cracks and intersections observed on the surface of PU admixed samples were both higher than that of natural soil, and could be up to 45 and 18, respectively (Figure 7b). By comparison, the *N*_n_ and *N*_c_ of PAM admixed soil both was smaller than that of natural soil, and decreased to 27 and 10 respectively (Figure 8b). The relationships between average length and average width of cracks and PU concentration (Figure 7c) suggest that the increasing PU concentration resulted in a significant increase of average length and slight increase of average width. The maximums of average length and average width of cracks generated on PU admixed samples were 23.7 mm and 13.9 mm respectively. Similar to PU, PAM concentration also had an acceleration effect on the average length and average width of cracks until it reached 0.075% (Figure 8c). The maximums of average length and average width of cracks generated on PAM admixed samples were 25 mm and 15.8 mm respectively. The soil clods number formed on PU admixed sample was decreased as PU concentration increases (Figure 7d), and fell between 22 and 29. For PAM admixed sample, the clods number also reduced, from 24 to 16 (Figure 8d). However, the average area of clods was enhanced with additives concentration. The maximum of average area of clods of PU-clayey soil mixture was 296.97 mm^2^ while the corresponding clods number was 22. Similarly, the maximum of average area of clods was 432.47 mm^2^ obtained from the PAM admixed sample with least clods number (i.e., 20). Actually, there was a negative correlation between clods number and the average area of clods when the area of the sample surface was constant.

The quantitative analyses on cracks show that as additives concentration increases, the crack propagated towards longer and wider, and the soil clods became bigger. In addition, the cracks number, intersections number, and clods number obtained from PU-clayey soil mixture were all greater than that of PAM admixed sample. These confirmed that the cracking and crushing degree of PU admixed sample was greater than that of PAM admixed sample. 

### 3.3. Characterization of Crack and Clod

**Figure 9:**
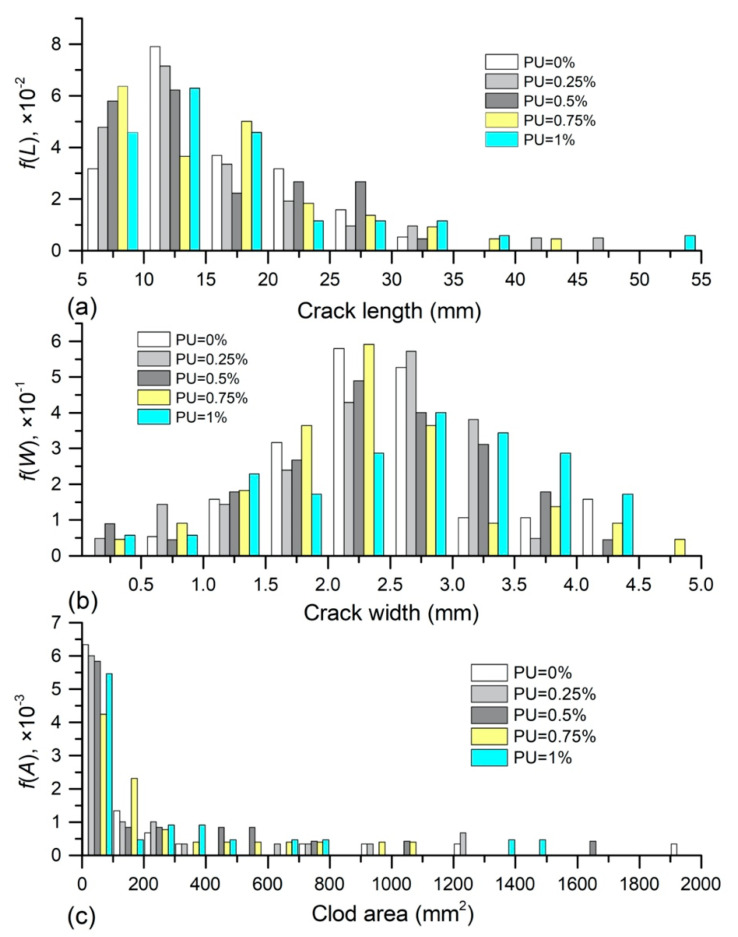
Probability density function of (**a**) crack length, (**b**) crack width and (**c**) clod area of PU admixed samples.

Probability density function of crack length *f*(*L*), crack width *f*(*W*) and clod area *f*(*A*) obtained from PU admixed samples is presented in Figure 9. Here, the crack length, crack width, and clod area corresponding to the maximum values of *f*(*L*), *f*(*W*), and *f*(*A*) are considered as the most probable value (MPV), suggesting the cracking situation which occurred most possibly during drying. It appears from Figure 9a that the crack length of all samples mainly distributed between 5 mm and 20 mm. A typical unimodal distribution of crack length was observed for the natural sample and PU admixed sample, while 0.75% PU admixed sample exhibited a bimodal distribution. Tang et al [25] attributed the presence of bimodal distribution to two different cracks formed on sample surface: primary long main crack and short sub crack. For reference sample, the MPV of *f*(*L*) was 12.5 mm; for PU admixed sample, it was 12.5, 12.5, 7.5, and 12.5 mm with PU concentration of 0.25%, 0.5%, 0.75% and 1% respectively. Whereas, the probability of longer crack generated on PU admixed sample was slightly higher in comparison with natural sample. Figure 9b shows that the distribution range of crack width slightly increased with PU concentration. For example, the crack width of natural soil was mainly distributed in the range of 1.5–3 mm, but the distribution range of 1% PU admixed sample was between 2 and 4 mm. Correspondingly, the MPV of *f*(*W*) also increased slightly as PU concentration increases. A typical unimodal distribution was also observed for the reference sample and PU admixed sample, except for the ones with PU concentration of 1%. In Figure 9c, it could be observed that the sample surface was split into small clods (the area was between 0 and 100 mm^2^) in majority and some large clods (the area was larger than 100 mm^2^), whatever the reference sample or PU admixed samples. However, the probability of a large clod formed on the PU admixed sample was slightly higher than that on the reference sample.

**Figure 10:**
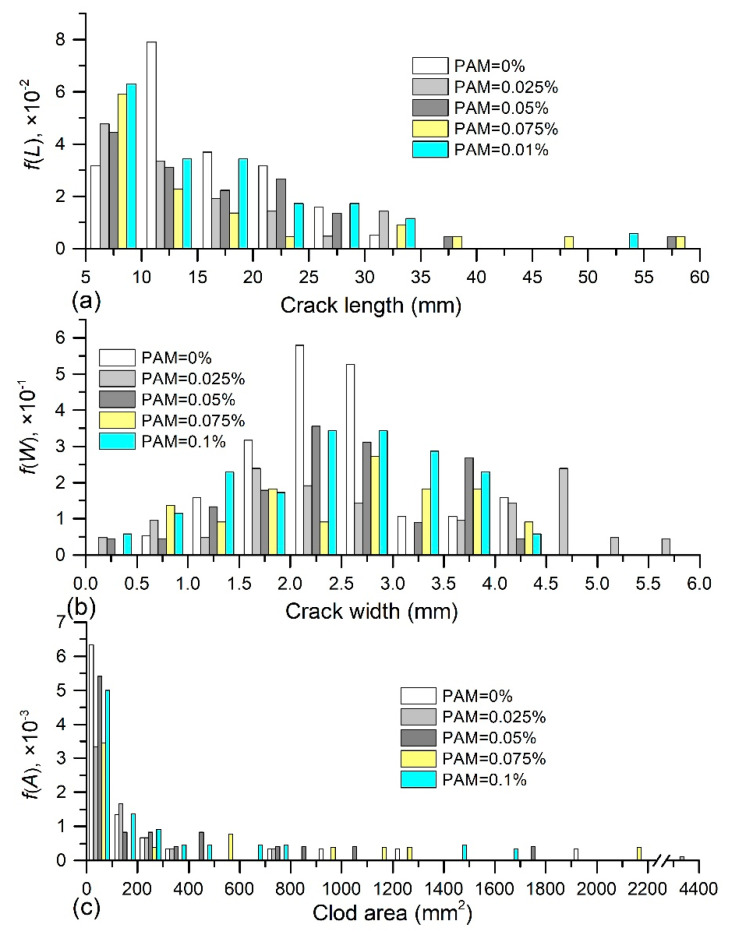
Probability density function of (**a**) crack length, (**b**) crack width and (**c**) clod area of PAM admixed sample.

Figure 10 displays the *f*(*L*), *f*(*W*), and *f*(*A*) obtained on PAM admixed samples. It could be observed that the crack length mostly fell in the range of 5 mm to 25 mm, and PAM addition had no significant effect on distribution range. However, the MPV of *f*(*L*) for admixed samples increased from 5 mm to 10 mm as PAM content increases, which was lower than that of reference sample. In addition, typical unimodal distribution was identified for reference sample and PAM admixed samples with concentration of 0.025% and 0.075%, while bimodal distribution was observed on the samples with PAM concentration of 0.05% and 0.1%. In Figure 10b, it shows that similar as PU addition, PAM addition also slightly enhanced the distribution range of crack width, and increased the possibility of occurring wider crack. Correspondingly, the MPV of crack width enhanced slightly from 2.25 to 2.75 mm. Different from the reference sample, bimodal distribution was observed on PAM admixed samples. Figure 10c presents that the split soil clods on PAM admixed sample were composed of dominant small clods (the area was between 0 and 100 mm^2^) and some large clods (the area was larger than 100 mm^2^), which was similar to the PU admixed sample. Also, the probability of forming large clods on PAM admixed sample was slightly higher. 

### 3.4. Microstructure Characteristics

**Figure 11:**
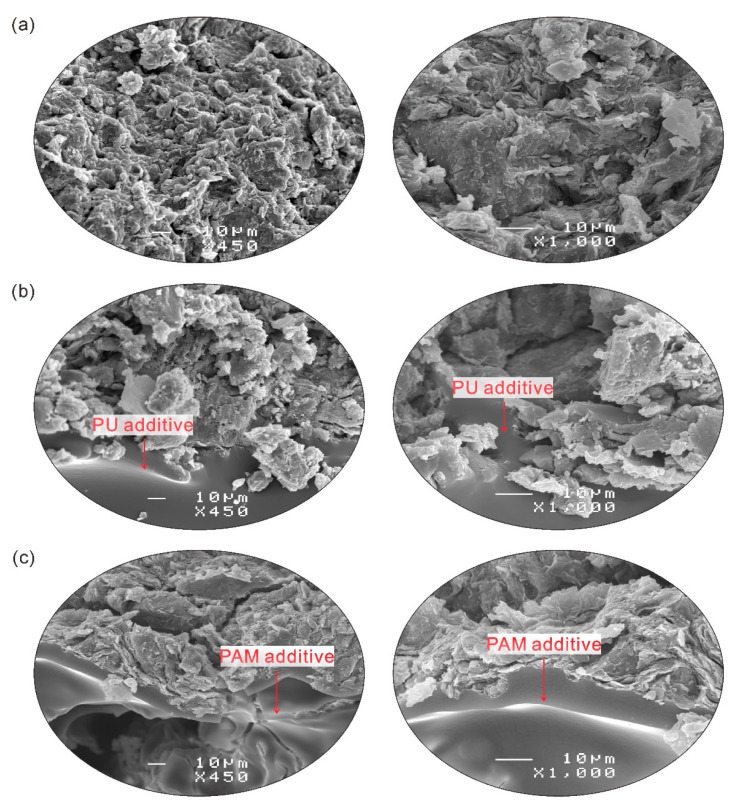
The SEM images of (**a**) reference sample, (**b**) PU admixed sample and (**c**) PAM admixed sample.

The SEM images obtained from natural clayey soil, 0.5% PU and 0.05% PAM admixed samples after drying are presented in Figure 11a–c, respectively. For the reference sample, it is observed that there was a porous and discontinuous layered texture in the surface (Figure 11a). Besides, the soil aggregates were scattered on the surface due to poor connection between them. In Figure 11b, it exhibits that PU additive penetrated through the inter-aggregates pores and distributed on the surface of aggregates, and bonded them together. It has been reported that hydrogen bonds and covalent bonds could be established between the soil grains and the PU molecule when the PU was evenly distributed on the surface of soil grains [8,44]. Figure 11c shows the smooth surface morphology that was formed by the PAM molecule enwrapped the surface of aggregates and formed a complete membrane structure on the surface. In general, the anionic PAM adsorption occurs through the polyvalent exchangeable cations bridging and electrostatic attraction [29,45]. Also, the degree of such absorption depended on the type and number of exchangeable cations, amount of clay, pH, and polymer molecular size [29,46]. 

**Figure 12:**
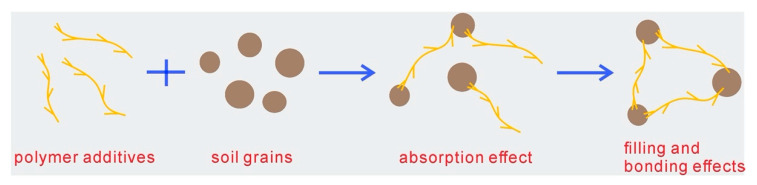
The effects of polymer additives on soil grains and microstructure.

Therefore, the effects resulted from PU and PAM addition on soil microstructure could be summarized as absorption, filling, and bonding effects, as shown in Figure 12. Actually, except for the additives adsorbed on the surface of aggregates, the penetration of additives through the inter-aggregates pores as well as its existence at pores reduced the voids to a certain extent. Soltani-Jigheh et al. [47] found that the mean diameter of pore of fine-grained soil decreased from 8.43 nm to 8.33 nm and the pore volume decreased from 16.026 cm^3^/g to 15.605 cm^3^/g due to the PAM addition according to BET results. For the admixed samples, the presence of polymer also created a great bonding between aggregates, which subsequently increased the aggregates size and enhanced the connection strength and stability. It was observed that the specific surface area of fine-grained soil reduced from 70.12 m^2^/g to 68.07 m^2^/g because of the presence of PAM [47]. Moreover, the fact that the PU and PAM polymer both produced a long chain that interlinked soil grains together and enhanced the soil resistance against shear forces, erodibility, and collapse has been indicated in the laboratory [13,14,37,48]. 

## 4. Discussion

Desiccation cracking is a complicated natural phenomenon for soil, mainly induced by water loss, that is, evaporation is a precondition for desiccation cracking. In the natural state, soil is a kind of porous medium with water in its pores. For clayey soil, due to isomorphic replacement, secondary mineral dissociation or selective adsorption, the surface of clay particles generally has negative charges, which are compensated by cations adsorbed on the crystal layer surface. Under the hydration of cations, the surface of clay particles is surrounded by a layer of bound hydration membrane. Due to the existence of hydration membrane, the clay particles do not directly contact with each other, but have spacing, which provides a lot of space for the shrinkage of cohesive soil. In the process of evaporation, evaporation always starts from the surface of soil. The first loss is the free water between soil particles. With continuous evaporation, capillary water will be produced in the soil, which makes the water in the lower soil transfer to the upper layer to maintain evaporation, and the water content of the soil decreases continuously. The distance between soil particles also gradually decreases, which leads to the formation of tensile stress field between soil particles. Due to the heterogeneity of soil, the complexity of composition, structural differences, and environmental factors, the distribution of tensile stress field is uneven, which makes it easy to cause stress concentration in the weak position of soil particle connection, and then form cracks. The factors affected the soil evaporation process could be concluded as external factors in terms of air flow rate, relative humidity and temperature, and inner characteristics of soil in terms of soil composition, pore structure, size distribution of soil grains and soil layer thickness [49,50,51]. 

**Figure 13:**
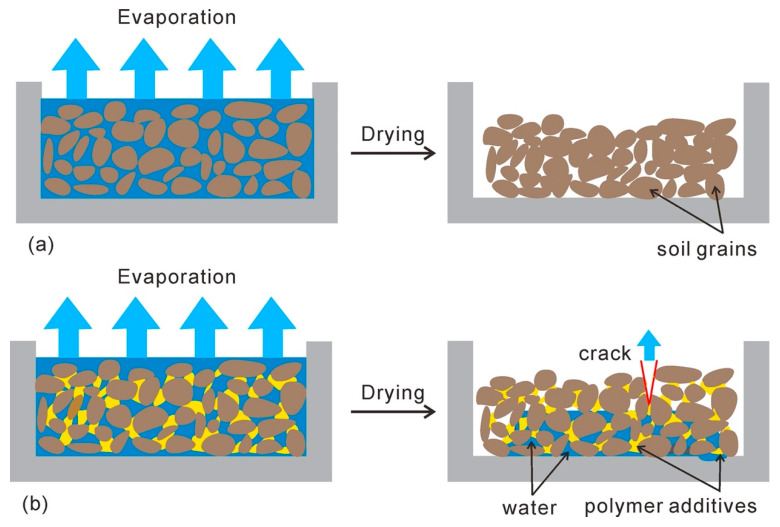
The schematic diagram of evaporation of (**a**) natural soil and (**b**) polymer admixed soil [41].

In the current study, the external condition was consistent for all samples with different states. The SEM observations show that the presence of additives led to changes on pore structure for a given soil. In general, the PU and PAM dissolve in water could adsorb a large amount of water and swell, finally exhibit as hydrogel [52,53]. Their presence at the inter-aggregates voids really reduced the pore space to a certain extent. The schematic diagram of soil evaporation is displayed in Figure 13. As shown, the presence of polymer additives influenced the formation of an effective water migration channel, and thus slowed down the evaporation rate [41]. As shown in the Figure 3, Figure 4 and Figure 5, the presence of PU and PAM significantly slowed down the evaporation rate and prolonged the duration of the constant evaporation stage. Moreover, in comparison with natural soil (Figure 13a), the presence of PU and PAM hydrogels enhanced the water hold capacity of admixed samples, which might be another reason for the decline of evaporation rate of admixed samples. In contrast, for the admixed samples with a higher concentration, it was prone to form cracks at the upper layer because the blocking effect caused by additives prevented water migration from the bottom to upper, and the cracks subsequently became another evaporation surface to accelerate the water evaporation (Figure 13b). This might be the key reason why the presence of PU and PAM accelerated these samples entering into the residual evaporation stage (sees Figure 3 and Figure 4). 

The factor that affected the development of tensile stress field between soil particles would affect the behavior of desiccation cracking [19]. In general, the PU and PAM addition both enhanced the tensile strength between soil aggregates via its absorption and bonding effects, which in turns could delay and prevent the increase of surface crack. However, the bonding effect of polymer usually worked well when it was in “dry” state, that is, when the additives transformed from a “rubbery” state to a “glassy” state by water evaporation [54]. In this study, the initial water content was 60%, which is far beyond the liquid limit of the experimental clayey soil. A large amount of water in the sample makes the polymer in a “rubbery” state for a long time, and cannot be transformed into “dry” state in a short time. Therefore, the polymer additives existed in the form of hydrogel at initial period would result in that the roles of polymer were not fully gave into play; this might be the main reason why the polymer had no significant effect on cracking behavior. Nevertheless, PAM reduced the cracking and crushing degree of soil to a certain extent. The addition of PAM reduces the number of cracks by 1%–4% and increases the average area of clods by 50 mm^2^ to 250 mm^2^, which could be seen in Figure 8. However, the changes of cracking behavior had no good regularity with PAM concentration, which could be explained by the fact that the distribution density and distribution location (on the inter-aggregates voids or on the surface of aggregates) was influenced by the higher consistency and viscosity of PAM, and this in turn affected the effectiveness of polymer addition. However, for PU admixed samples, it was observed that the crack intensity factor was slightly higher than that of natural soil, and it increased from 19.12% to 20.53% with different concentrations. It also increases the number of cracks by 1–2%, and the average area of clods by 10 mm^2^ to 75 mm^2^, which can be seen in Figure 7. This might be resulting from the fact that, in addition to the water absorbed by clay mineral, the water absorbed by PU additives also provided space for soil shrinkage during drying [43]. This might be the main reason for the width of crack increase. Thus, necessary attention should be paid to the desiccation cracking of polymer treated soil when applied in the field. 

## 5. Conclusions

A series of desiccation tests were conducted on polyurethane and polyacrylamide admixed clayey soils to investigate the effect of polymer addition on desiccation cracking behavior. The water evaporation, crack initiation, and propagation processes were recorded and analyzed. The microstructure changes were also studied using SEM images. According to the results obtained, the following conclusions could be drawn:

(1) The soil evaporation process could be affected by polymer. PU and PAM addition both decreased the evaporation rate of initial stage might by storing water in voids and filling inter-aggregates voids, about 12%, and the effects were related to polymer dosage. However, the polymer addition with higher dosage accelerated water evaporation and shortened the duration of the water loss process by forming cracks due to the blocking effects caused by additives. 

(2) Final cracks morphology analyses of polymer admixed soils show that PAM addition slightly reduced the cracking and crushing degree and kept the soil relatively intact, also reducing the number of cracks by 1–4% and increasing the average area of clods by 50 mm^2^ to 250 mm^2^. However, PU addition slightly enhanced the cracking and crushing degree of soil, and increases the number of cracks by 1–2%, and the average area of clods by 10 mm^2^ to 75 mm^2^. In addition, PU and PAM addition both could increase the width and length of cracks. 

(3) The polymer created a great bonding between soil aggregates and enhanced soil resistance to cracking. However, it was observed that polymer had no significant effect on cracking behavior. This might because the existence of the hydrogel form led to the bonding effect to be insufficient. Additionally, the water absorbed by polymers also provided space for soil shrinkage during drying, and thus slightly enhanced the crack intensity factor and increased the crack width.

In this study, only the evaporation and cracking processes of soil under the condition of single initial water content were considered. Meanwhile, the polymer concentration was relatively low. In the follow-up study, the case of multiple initial water content and high polymer concentration will be fully considered. In addition, the difference of probability density function of different variables will be also the focus of follow-up research.

## Figures and Tables

**Table 1:** Physical properties of used clayey soil.

Item	Value
Specific gravity	2.73
Consistency limit	
Liquid limit (%)	36.7
Plastic limit (%)	18.9
Plasticity index	17.8
USCS classification	CL
